# Pressurized liquid extraction of glucosinolates from *Camelina sativa* (L.) Crantz by-products: Process optimization and biological activities of green extract

**DOI:** 10.1016/j.fochx.2024.101324

**Published:** 2024-03-24

**Authors:** Stefania Pagliari, Gloria Domínguez‐Rodríguez, Alejandro Cifuentes, Elena Ibáñez, Massimo Labra, Luca Campone

**Affiliations:** a Department of Biotechnology and Biosciences, University of Milano-Bicocca, Milan, Italy; b NBFC, National Biodiversity Future Center, 90133 Palermo, Italy; c Foodomics Laboratory, Instituto de Investigación en Ciencias de la Alimentación (CIAL, CSIC-UAM), Nicolás Cabrera 9, Campus de Cantoblanco, 28049 Madrid, Spain

**Keywords:** Glucohirsutin (PubChem CID 44237258), Glucoarabin (PubChem CID 46173881), Glucocamelin (PubChem CID 162639109), Pressurized liquid extraction, *Camelina sativa* by-products, Glucosinolates, Bioaccessibility

## Abstract

The cultivation of *Camelina sativa* (L.) Crantz is rapidly increasing due to oil production resulting in a substantial volume of by-products, which still have an interesting composition in secondary metabolites, especially glucosinolates. Therefore, a green extraction procedure of glucosinolates by Pressurised Liquid Extraction was developed and optimized using a chemometric approach. Furthermore, the glucosinolates were purified by solid phase extraction, and a preliminary study on bioaccessibility and bioavailability study was carried out to evaluate the resistance of the glucosinolates to the digestive process. The application of pressurised liquid extraction to the recovery of glucosinolates from camelina sativa by-product, is a green, automatic, and rapid method, representing a valid alternative to conventional extraction method to obtain ingredients for food industries.

## Highlights

• The Camelina sativa by-products as source of glucosinolates was explored.
• A higher bioactive content can be obtained by unconventional developed method.
• Optimization of extraction parameter by response surface design.
• Glucohirsutin were identified by UPLC-UV-HRMS/MS for the first time in Camelina sativa.

## 1 Introduction

*Camelina sativa* (L.) Crantz is an herbaceous plant in the Brassicaceae family that has received great attention due to its high tolerance to different abiotic factors and its ability to grow under unfavourable conditions (Blume et al., 2023), its cultivation for food and feed purpose is rapidly increasing in Europe and North America. *C. sativa* seeds contain 30–40 % oil rich in essential fatty acids, which can be an excellent plant-based alternative to fish and flaxseed oil and high levels of bioactive molecules such as tocopherol, phytosterol, and phenolic compounds (Ergönül and Özbek, 2020, Gugel and Falk, 2011, Piravi-vanak et al., 2022). Oil production generates camelina pressed-cake (PC) by-product, requiring disposal. Unfortunately, this often leads to severe environmental issues due to inadequate management. It is well documented that the PC has an interesting chemical composition (amino acid and protein, glucosinolates, synapine, inositol phosphates, tannins, and erucic acid) and could be reused in different industrial applications (Erickson and Klopfenstein, 2012, Zubr, 1997). Glucosinolates (GLSs) are secondary metabolites particularly abundant in the Brassicaceae family; they consist of a d-thioglucose group linked to a sulfonated aldoxy group and a variable side chain derived from an amino acid. Nowadays, more than 130 different glucosinolates have been identified in many plants, especially in the Brassicaceae (Thinh Nguyen et al., 2020, Xin et al., 2014). GLSs composition and concentration vary based on species, variety, environment conditions, plant age. (Fahey et al., 2023, Mithen, n.d., Ram Bhandari et al., 2015, Pagliari et al., 2022a). Recent studies have associated GLSs with several health benefits such as antioxidant (direct and indirect), anti-inflammatory, antimicrobial, cholinesterase inhibitory, anti-tumor, and cardiovascular disease preventive properties (Maina et al., 2020, Sánchez-Pujante et al., 2017). In this context reusing the PC via extraction processes can generate potential products for the food pharmaceutical, nutraceutical, and cosmetic industries.

Usually, the recovery of secondary metabolites from agricultural by-products requires several time-consuming and potentially hazardous steps, involving the use of toxic extraction solvents. It is therefore necessary to use sustainable processes with the lowest cost and environmental impact, to extract these metabolites from PC, quickly, efficiently, and selectively (Belwal et al., 2018, Amran et al., 2021). Among the extraction processes pressurised liquid extraction (PLE) is a highly efficient techniques compared to the traditional extraction methods. PLE involves the use of liquid solvent at an elevated temperature and pressure to enhance the extraction of target compounds from the matrix. The combination of pressure and temperature increase the mass transfer rate by reducing the surface tension and viscosity of the solvent (Mustafa and Turner, 2011, Figueroa et al., 2018, Pagano et al., 2021). This simultaneously increases the solubility of the analytes facilitating the penetration of the solvent into the matrix. PLE has many advantages over conventional extraction techniques (maceration, distillation, Soxhlet, etc.), such as reducing energy consumption, waste management, production time, and costs, while improving consumer safety and health, regulatory compliance, and preserving bioactive compounds in the matrix (Figueroa et al., 2018, Pagano et al., 2021).

In the present work, a PLE method was used to recover glucosinolates from camelina PC. Initially, an analytical procedure based on ultra-pressure liquid chromatography (UPLC) coupled with high-resolution mass spectrometry (HRMS) was developed to evaluate the chemical composition of the extract. Furthermore, a response surface design was used for the identification of experimental parameters that significantly influenced the extraction as well as the interaction between these parameters.

Under the optimized extraction conditions, the developed PLE method was compared with conventional methods such as ISO and USAE (Pagliari et al., 2022a) showing better efficiency, with a reduction of time and solvent consumption. Finally, the biological activities of the GLSs identified in PC, which are still poorly known, were investigated using in silico and in vitro assays to identify their potential beneficial activity for human health. Considering the promising chemopreventive activities and the dietary use of the species, an initial bioaccessibility and bioavailability study was initiated to assess the resistance of the GLSs to the digestive process and to provide information for a suitable formulation to preserve their properties.

## 2 Materials and methods

### 2.1 Standards and materials

MS-grade solvents used for UPLC analysis acetonitrile (MeCN) water (H_2_O) and formic acid (HCOOH) were provided by Romil (Cambridge, UK); analytical-grade solvents methanol (MeOH) and ethanol (EtOH) were supplied by Sigma-Aldrich (Milan, Italy). H_2_O was purified by using a Milli-Q system (Millipore, Bedford, USA). Acetic acid (AA), ammonium hydroxide, naphthylethylene diamine dihydrochloride, phosphoric acid, ascorbic acid, fluorescein sodium salt, Trizma hydrocloride (Tris-HCl) monopotassium phosphate dipotassium phosphate, sodium nitroprusside dehydrate (SNP), sulphanilamide were provided by Sigma-Aldrich (Milan, Italy). 2,2-azobis(2-amidinopropane) dihydrochloride (AAPH) were purchased from TCI Chemicals (Tokyo, Japan). Glucoarabinin potassium salt, glucocamelin potassium salt and homoglucocamelinin potassium salt were purchased from Extrasynthese (Lyion, France).

### 2.2 Samples

The industrial PC were received after cold oil extraction from FlaNat Research srl (Milan Italy). The samples was finely blended using a knife mill, Grindomix GM-200 (Restek GmbH Germany) operated at 6000 rpm (Pagliari et al., 2022b). The ground sample was sieved to obtain a powder with a homogeneous particle size distribution and fraction retained on the 300–600 μm mesh was collected and stored in the dark at −80 °C in polyethylene bags until used in the extraction processes.

### 2.3 Pressurized liquid extraction (PLE)

The ASE300 was used for pressurized liquid extractions (Thermo Fisher, Waltham, USA). For the extraction, 1 g of dried material was packed into a 10 mL stainless steel extraction cell and the extraction cell's empty area was filled with 4 mm solid-glass beads (Sigma-Aldrich, Milan, Italy), and a paper filter (Whatman n°1) was placed at the bottom of the extraction cell.

Preliminary tests were performed with EtOH 80 %, 3 cycles, and static of 4 min to identify the extraction temperature range to be used in the experimental design. The extracts were collected in a glass vial (60 mL) and the solvent was evaporated using a rotary evaporator (G3, heiVAP core, Heidolph Germany) to calculate the extraction yield.

### 2.4 Optimization of PLE condition by an experimental design

Chemometric approach was used to find the best PLE extraction condition using Statgraphic Centurion XVI 16.1 version (Rockville, USA.). A response surface was employed to investigate the effects of four independent variables (extraction temperature, number of cycles, solvent composition, static time) on the dependent variable (mg/gDM of GLS9, GLS10, GLS11). A Box-Behnken design 2-factor interaction with 3 canter point, an error of 12 degree of freedom, for a total of 27 randomized run was used (Table 1). The four independent variables were examined at three levels (low, medium, and high) using this design: extraction temperature (Temp) at 70, 110, and 130 °C, number of cycles 2, 4, and 6, composition of solvent modifier (EtOH%) at 60, 80, and 100 %, and static time 2, 4, and 6 min.

**Table 1:** Experimental condition of the response surface design and experimental quantitative value of the response variable (GLS9, GLS10 and GLS11).

Run	EtOH(%)	Cycles(n°)	Temp(°C)	S.time (min)	GLS 9 (mg/gDM)	GLS 10 (mg/gDM)	GLS 11 (mg/gDM)
**1**	100	6	100	4	3.9	7.8	2.7
**2**	80	4	130	2	7.5	15.3	4.0
**3**	80	4	100	4	8.9	17.5	4.1
**4**	100	2	100	4	5.1	10.8	3.1
**5**	80	6	130	4	6.6	12.8	3.9
**6**	100	4	100	2	5.0	10.7	3.0
**7**	80	4	70	6	8.7	18.3	4.0
**8**	60	4	130	4	8.6	16.2	4.1
**9**	80	6	100	2	10.3	21.8	4.7
**10**	60	4	70	4	6.4	13.5	3.7
**11**	100	4	130	4	6.7	11.9	3.6
**12**	60	4	100	6	7.5	14.2	3.4
**13**	80	2	70	4	7.9	15.7	4.0
**14**	80	2	130	4	7.6	16.3	3.4
**15**	60	4	100	2	7.7	15.2	3.7
**16**	80	2	100	2	8.2	15.2	3.9
**17**	100	4	100	6	4.4	8.8	2.8
**18**	60	6	100	4	11.6	22.1	4.7
**19**	80	4	70	2	6.7	14.4	3.4
**20**	80	6	70	4	7.0	14.2	3.5
**21**	80	4	100	4	7.4	14.8	3.6
**22**	60	2	100	4	5.5	10.4	2.7
**23**	80	2	100	6	9.4	19.1	4.4
**24**	80	4	130	6	6.5	12.7	3.5
**25**	80	6	100	6	10.1	21.9	4.4
**26**	80	4	100	4	8.7	16.0	4.0
**27**	100	4	70	4	3.1	6.4	2.2

To get the parameters of the statistical models, data from the CCD were submitted to regression analysis using least square regression methods. ANOVA was used to determine the statistical significance of independent variable (A, B, C, and D) contributions and their first order interaction. The answers obtained from the statistical analysis were fitted to a second-degree model capable of taking into account the individual parameter interactions together with their quadratic relationships (eq (1)).

(1) γ = β_0+∑_(j = 1)^k β_j x_j+∑_(j = 1)^k β_jj x_j^2+∑_(j = 1)^k.∑_(j = 1)^k β_ij x_i x_j

### 2.5 Purification of glucosinolates by solid phase extraction

To obtain a GLSs rich extract the solid-phase extraction (SPE) was carried out based on our previous study (Pagliari et al., 2022a) with slight modification. Briefly, a strong anion exchange (SAX) Mega Bond Elute NH_2_ cartridges (1 g) were activated with 10 mL of MeOH and equilibrated with 10 mL of H_2_O 1 % AA. The PLE liquid extract was loaded onto the NH_3_^+^ cartridge, washed with 5 mL of MeOH 1 % AA; finally, the glucosinolate fraction was eluted with 10 mL of freshly prepared H_2_O 2 % NH_4_OH solution. The purified extract was evaporated to dryness in a vacuum evaporator at 40° C, dissolved in water at a concentration of 1 mg mL^−1^ and filtered with 0.22 µm PES filter before in vitro assays.

### 2.6 Comparative analysis of extraction techniques

An extraction technique previously developed was used to compare the performance of the developed PLE method. The ultrasound-assisted extraction (USAE) was developed and optimised in our previous study (Pagliari et al., 2022a). Briefly, 1 g of ground samples was extracted 2 times with 5 mL of 65 % EtOH in in the ultrasonic bath (Sonorex TK 52; Bandelin electronic, Berlin, Germany) operating at 35 kHz and power, 100 %. All the extraction were performed in triplicate, centrifuged (ALC centrifuge PK 120, Thermo Electro Corporation, San Jose, CA, USA) for 3 min at 13000 rpm (19.8 g). The supernatants were filtered with a paper filter (Whatman No. 1 filter) and stored at −20 °C until the analysis by UPLC-HRMS.

### 2.7 Qualitative and quantitative analysis by HRMS/MS analysis

Qualitative and quantitative analyses of extracts were carried out using an acquity UPLC system coupled with a Xevo G2-XS QTof mass spectrometer (Waters Corp., Milford, MA, United States). The mass spectrometer equipped with an electrospray ion source (ESI), was used in negative and positive ionization modes to acquire full-scan MS, and spectra were recorded in the range of 50–1000 *m*/*z*. The ESI parameters were as follows: electrospray capillary voltage 2.0 kV, source temperature 150 °C and desolvation temperature 600 °C. The cone and desolvation gas flow were 20 and 900 L h^−1^, respectively, and a scan time of 0.3 s was employed. Cone voltage was set at 70 V, and source offset at 20. The mass spectrometer was calibrated with 0.5 M sodium formate, and 100 pg µL^−1^ of standard leucine-enkkephaline at *m*/*z* 554.2615 was infused with the flow of column at 5 µL min^−1^ as the lock mass and acquired for 1 s each 30 s. The total ion current (TIC) used for qualitative analysis was acquired, and MS/MS spectrum of each compound at different collision energy was acquired and compared to reference standards from which the GLSs identification was performed. A quantitative analysis was performed using multiple reaction monitoring (MRM) data acquisition mode by monitoring three characteristic fragments for each target compounds of [M + H]- ion of glucoarabinin (506.1523 > 442.14, 248.11, 96.96) glucocamelinin (520.1684 > 456.16, 262.12. 96.96) and homoglucocamelinin (534.1819 > 470.18, 276.14, 96.96) and ramping collision energy from 25 to 30 V to produce abundant product ions before the detection. In order to quantify the GLS compounds in the extracts, an external standard calibration was conducted at six points between 0.01 and 10 μg mL^−1^. Each level was acquired in triplicate. The analysis of variance (ANOVA) was carried out to test the regression curves, and the linear model was found appropriate over the concentration range (R^2^ values > 0.9992). Precision and intraday repeatability were also estimated in all the concentration levels with a coefficient of variation lower than 5 %. The results of the quantitative analysis for each analyte were expressed as mg g^−1^ of dry matter (DM). The Mass Lynx software (version 4.2) was used for instrument control, data acquisition, and processing.

### 2.8 In vitro antioxidant activities

#### 2.8.1 ABTS assay

ABTS assay was used to evaluate the antioxidant activity of all sample extracts. The experimental conditions were reported by Pagano et al. (Pagano, Sánchez-Camargo, et al., 2018). Briefly, 5 mL of PBS (control), Trolox (0.25–1 mg mL-1) and extracts 1 mgmL-1 were mixed with 500 mL of ABTS standard solution at a concentration of 1 mM. 300 mL of each mixture were transferred into a 96 well plate and were incubated, protected from light, and after 60 min the absorbance was read at 734 nm using a Multiskan Go spectrophotometer (Thermo Fischer Scientific). Results of ABTS assay were expressed as Trolox equivalent TEAC mmol/mg, and they were employed to quantify the antioxidant activity of the tested solution expressed as standard deviation (SD) of three measurements.

#### 2.8.2 Oxygen radical absorbance capacity (ORAC)

The scavenging capacity of extracts against oxygen radicals was evaluated using the ORAC assay. The experiment is performed in black 96-well plates according to the protocol reported by Sánchez-Martínez et al. (Sánchez-Martínez et al., 2022). Each well is filled with 100 µL of extract at different concentrations in water, 100 µL of AAPH (590 mM) in 30 mM phosphate buffer saline (PBS) at pH = 7.5, 25 µL of fluorescein in PBS and 100 µL of PBS to make up the volume. Fluorescence was measured with λ excitation = 485 nm and λ emission = 530 nm every 5 min for 1 h at 37 °C. Ascorbic acid was used as a positive control. The peroxyl radical scavenging ability of the extract was expressed as IC50 (µg of extract able to inhibit 50 % of the AAPH radical).

#### 2.8.3 RNS scavenging capacity

RNS radical scavenging capacity was measured by a point spectrophotometric assay capable of measuring the ability of the sample to neutralize nitric oxide (NO) radicals. The assay was prepared according to Sánchez-Martínez et al. (Sánchez-Martínez et al., 2022). The assay was performed in transparent 96-well plates. Each well was filled with 100 µL of the extract at different concentrations in water and 50 µL of SNP (5 mM) solubilized in 30 mM PBS at pH = 7.5. After incubation for 2 h under the white light of a lamp at room temperature, 100 µL of Griess reagent (prepared by mixing 500 mg sulfanilamide with 50 mg naphthyl-ethylenediamine dihydrochloride and 1.25 mL phosphoric acid in 48.5 mL water) was added. After incubation for 5 min, the absorbance at 734 nm was recorded to measure the concentration of nitrite radicals. Ascorbic acid was used as a reference standard. The results was expressed as IC50 (µg of extract able to inhibit 50% of SNP radical).

### 2.9 In vitro gastrointestinal digestion simulation

Gastrointestinal digestion was carried out following the INFOGEST protocol described by Minekus (Minekus et al., 2014), with light modification by Pagliari (Pagliari et al., 2023). Briefly, the oral, gastric, and intestinal phases were simulated by mimicking the salt and enzyme composition, pH, time, and temperature conditions of each phase of the digestive process. Specifically, in the oral phase, 2 mL of PC extract was added to 1.4 mL of oral saline (SSF), 390 µL of water, 200 µL of amylase 75 U/mL and 10 µL of CaCl_2_ 0.3 M. After 2 min at 37 °C, 3 mL of gastric saline (SGF), 640 µL of pepsin 2000 U/mL, 258 µL of water and 2 µL of CaCl_2_ 0.3 M and 160 µL of HCl 4 M were added to the 4 mL of oral phase to achieve a pH between 2 and 3. After 2 h at 37 °C, the final intestinal phase was started by adding 4.4 mL intestinal salt phase (SIF), 2 mL porcine pancreatin to obtain trypsin at 100 U/mL, 14 mg porcine bile, 1.58 mL water and 16 µL CaCl_2_ 0.3 M. The pH was maintained at 8 during the intestinal phase (2 h at 37 °C). At the end of the process, the digested extracts were analysed by UPLC-HRMS to quantify the GLSs compounds. The Bioaccessibility and bioavailability were calculated according to Eq. (1) and Eq. (2) respectively end expressed as percentage as reported by Cañas et al. (2022)

(1) $$\%bioaccessibility=\frac{\mathrm{digestedfraction}}{\mathrm{non}-digestedfraction}*100$$

where non-digested fraction and digested fractions are the concentration of compound before and after digestion simulation, respectively.

(2) $$\%bioavailaility=\frac{\mathrm{digestedfraction}*absorption}{\mathrm{non}-digestedfraction}*100$$

The absorption was estimated in silico using pkCSM-pharmacokinetics (https://biosig.unimelb.edu.au/pkcsm/, consulted on 5 September 2023) e ADMETlab (https://admet.scbdd.com/, consulted on 5 September 2023).

### 2.10 Statistical analysis

All data were performed in triplicate and results were presented as average ± standard deviation. Analysis of variance (ANOVA) was used to compare the means while Turkey’s test was used to assess the statistically significant difference among extraction conditions using JMP 14 software. A p-value of ≤ 0.05 was considered significant.

## 3 Results and discussion

### 3.1 Analysis of PLE extract

Although the characterization of the PC extract obtained by USAE had already been reported in our previously work (Pagliari et al., 2022a), we carried out further analysis using UHPL-HRMS/MS on the PLE extract to verify the stability of its chemical composition under the combined influence of temperature and pressure. Indeed, the temperatures and pressures employed in PLE can modify the chemical composition of extract and may lead to the degradation of molecules when compared to USAE. For this reason, an extraction under mild conditions (70 °C, two extraction cycles and 65 % EtOH) was performed to investigate the preliminary chemical composition of the PLE extract. Identification of compounds were assigned by using all chemical information; retention times (Tr), UV/vis signals, accurate mass, molecular formula, MS/MS spectra, reference standards whenever available, combined with chemo-taxonomic databases. The detailed characterisation of the phytochemical compounds selected condition was performed using UPLC-HRMS and the obtained results were in agreement with those reported in our previous study (Pagliari et al., 2022a). In detail as reported in Figure S1, the untargeted analysis in negative ion mode reveals the presence of 11 main metabolites, including phenols (epicatechin-O-glucoside isomer (1 and 2), rutin-2-O-apioside (4), rutin (6), Kaempferol-3-O-gentiobioside-7-O-rhamnoside (7), Kaempferol-3-O-neohesperidoside (8), Isorhamnetin-3-O-β-rutinoside (9), Tamarixetin-7-O-rutinoside(11) and three glucosinolates, glucoarabin (3) (GLS9), glucocamelinin (5) (GLS10) and homoglucocamelinin (10) (GLS11).

### 3.2 Optimization of glucosinolates extraction by PLE

#### 3.2.1 Preliminary selection of solvent composition and temperature

**Fig. 1:**
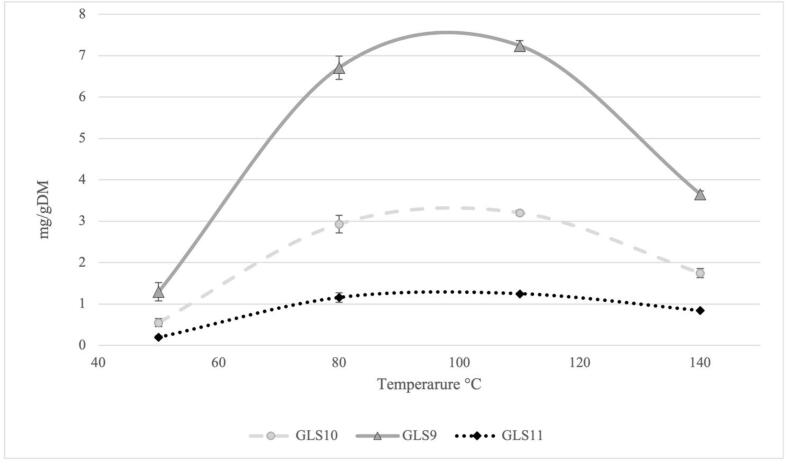
Effect of the temperature on of the three GLSs yield (mg/g DM) in PLE extracts.

Once the presence of GLSs in PLE extract has been confirmed through qualitative analysis, the extraction conditions were optimised using chemometric approach. As generally reported in the literature, the parameters that most influence the PLE process are temperature and solvent composition (Pagano, Sánchez-Camargo, et al., 2018). Preliminary experiments were carried out to determine the ranges of temperature and solvent composition to be used before chemometric optimization. The effect of PLE temperature on extraction efficiency of GLSs was showed in Fig. 1. The results show a direct correlation between the rise of PLE temperature and the extraction yield of GLSs up to a temperature of 110 °C, beyond which the recovery of glucosinolates starts to decrease, probably because of the degradation of these compounds. Indeed, it is known that GLSs are thermosensitive molecules and most of them could be degraded over at 100 °C (Hanschen et al., 2012, Oerlemans et al., 2006). Based on these results, the temperature range of 70–130 °C was used in the chemometric optimization. Regarding the solvent composition, EtOH was chosen as Generally Recognized as Safe (GRAS) solvent for safe food use and for its efficiency in the recovery of GLSs, as previously demonstrated in USAE (Pagliari et al., 2022a). As demonstrated for the USAE extraction, waters produce a swelling of matrix due to the presence of saponins and mucilage in the camelina seeds, causing the clogging of the PLE system preventing the emptying of extraction cell, for this reason the minimum EtOH content to ensure the proper functioning of the PLE is EtOH 60 %. Following these results, the solvent composition range was set between 60 % and 100 % EtOH.

#### Response surface design of PLE process

After conducting preliminary experiments, to determine the temperature and solvent composition ranges, a Box-Behnken two-factor interaction design was employed to investigate the effect of four independent variables (temperature, number of cycles, static time and percentage of ethanol) on the dependent variable (the extraction efficiency of the three glucosinolates). Table 1 shows the experimental conditions for each run and the experimental values of the responses (GLS9, GLS10 and GLS11) to the different experimental conditions.

**Fig. 2:**
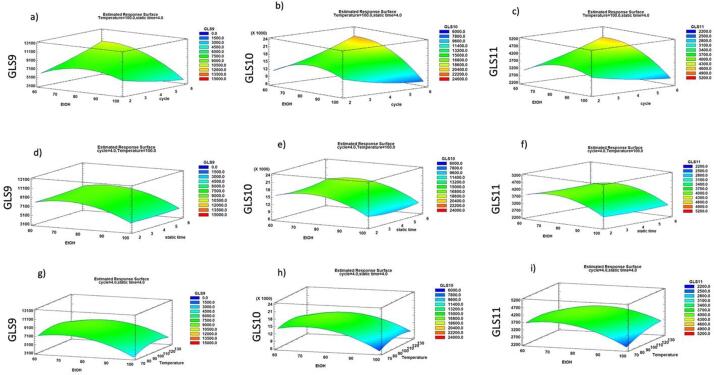
Response surface plots showing the effects of combination of %EtOH vs number of cycles (a-c); %EtOH vs Static time (d-f); %EtOH vs Temperature (g-i), on the recovery of glucosinolates.

The statistical analysis reported in Table 2 allows us to identify the significant factors and their impact on the response variables. Based on the results, the model showed a high correlation (R2 ≅ 77–79 %), indicating a low variance of the data and a good prediction of the model with respect to all the response variables considered. The obtained results demonstrate that only the percentage of ethanol and its combination with the number of cycles have a significant influence on the efficiency of the GLS9, GLS10, and GLS11 recovery (p-value < 0.05). The regression model equations that best describe the recovery of GLS as a function of process variables are shown in Table S1. As shown in the response surface (Fig. 2 a-c), the percentage of EtOH has a linear effect on the recovery of the three GLSs in the range from 60 to 65–70 %, above 65–70 % the desired effect decreases. This confirms the ability of PLE to use the combination of high temperature and pressure to significantly modify the chemical properties of the extraction solvent, improving the extraction efficiency of the water and reducing the necessity to use a high concentration of organic solvent. The number of cycles linearly increases the extraction efficiency when the EtOH percentage was low, whereas at high EtOH concentration increasing the number of cycles has a negative effect by reducing the recovery of the three GLSs (Fig. 2 d-f). The temperature follows a parabolic profile, indicating a peak after which recovery may decrease due to GLS structure degradation at high temperatures (MacLeod et al., 1981), while the extraction process was not affected by the static time. Temperature and static time were found to be statistically insignificant (Table 2). The optimized conditions extrapolated from DOE to maximize GLSs recovery were as follow 65 % EtOH, 6 cycles, 118 °C and 2 min static time (Opt 1), resulting in a desirability of 97.84 %. However, as the extraction temperature did not show statistical significance (p-value > 0.05) in the regression model, as the concentration of GLS9, GLS10, and GLS11 did not change significantly by varying the temperature, as shown in the response surface (Fig. 2 g-i), alternative extraction conditions were investigated. These conditions (Opt 2) were the same as adopted in Opt 1 but the extraction was conducted at 70 °C representing the minimum value tested in response surface) was tested. The results of conditions Opt1 and Opt2 indicate that there is no statistically significant difference (p-value > 0.05) between the two tested conditions. Therefore, Opt 2 was selected as a better solution to minimize energy consumption and to avoid the possible thermal degradation of GLSs, with an extraction yield of 148.08 ± 0.02 mg/g DM. Furthermore, the actual values, reported in Table 3, were in good agreement with the predicted values for the concentration of the three main glucosinolates by PLE, indicating the suitability of the established regression models for the prediction of response values.

**Table 2:** Analysis of variance of the regression model.

	Sum of squares			Mean squares		
	GLS9	GLS10	GLS11	GLS9	GLS10	GLS11
A:EtOH	3.01023E7	1.03447E8	2.07917E6	3.01023E7	1.03447E8	2.07917E6
B:cycle	2.86163E6	1.47054E7	468,470	2.86163E6	1.47054E7	468,470
C:Temperature	1.02726E6	617,894	249,697	1.02726E6	617894.	249,697
D:static time	122,614	520,833	2610.75	122,614	520833.	2610.75
A^2^	1.88627E7	8.3087E7	1.94837E6	1.88627E7.	8.3087E7	1.94837E6
B^2^	534,674	5.83389E6	54225.9	534,674	5.83389E6	54225.9
*C*^2^	4.5387E6	1.2012E7	133,001	4.5387E6	1.2012E7	133,001
D^2^	107,163	5.09212E6	18934.3	107163.	5.09212E6	18934.3
AB	1.32169E7	5.40666E7	1.35956E6	1.32169E7	5.40666E7	1.35956E6
AC	463,080	1.90026E6	213,444	463,080	1.90026E6	213,444
AD	44944.0	182329.	2862.25	44944.0	182329.	2862.25
BC	576.0	1.02414E6	245,025	576.0	1.02414E6	245,025
BD	475,410	3.51938E6	160,400	475,410	3.51938E6	160,400
CD	2.26804E6	1.06994E7	301,950	2.26804E6	1.06994E7	301,950
Total error	2.3288E7	8.9001E7	2.11423E6	1.94067E6	7.41675E6	176,186
Total (corr.)	1.02605E8	4.23083E8	9.94614E6	–	–	–
R^2^	77.3032	78.9637	78.7432	–	–	–
Adj R^2^	50.8237	54.4214	53.9437	–	–	–
	**F-value**			**P-value**		
	**GLS9**	**GLS10**	**GLS11**	**GLS9**	**GLS10**	**GLS11**
A:EtOH	15.51	13.95	11.80	**0.0020^a^**	**0.0028^a^**	**0.0049^a^**
B:cycle	1.47	1.98	2.66	0.2480	0.1845	0.1284
C:Temperature	0.53	0.08	1.42	0.4808	0.7778	0.2569
D:static time	0.06	0.07	0.01	0.8058	0.7955	0.9051
A^2^	9.72	11.20	11.06	**0.0089^a^**	**0.0058^a^**	**0.0060^a^**
B^2^	0.28	0.79	0.31	0.6092	0.3926	0.5892
*C*^2^	2.34	1.62	0.75	0.1521	0.2273	0.4020
D^2^	0.06	0.69	0.11	0.8182	0.4235	0.7487
AB	6.81	7.29	7.72	**0.0228^a^**	**0.0193^a^**	**0.0167^a^**
AC	0.24	0.26	1.21	0.6340	0.6219	0.2926
AD	0.02	0.02	0.02	0.8816	0.8780	0.9007
BC	0.00	0.14	1.39	0.9865	0.7167	0.2611
BD	0.24	0.47	0.91	0.6296	0.5040	0.3588
CD	1.17	1.44	1.71	0.3009	0.2529	0.2150

R^2 =^ Quadratic correlation coefficient. ^a^ Significant (p < 0.05).

**Table 3:** Quantitative analysis of GLS9, GLS10 and GLS11 between the optimal design condition suggested by software (Optimized 1) and the hypothetic optimal condition with the lowest energy consumption (Optimized 2).

GLSs	Predicted Optimized 1 mg g^−1^ DM	Optimized 1 mg g^−1^ DM	Predicted Optimized 2 mg g^−1^ DM	Optimized 2 mg g^−1^ DM
GLS 9	11.84^a^	12.4 ± 1.25^a^	9.25^a^	11.6 ± 1.15^a^
GLS 10	22.49^b^	22.98 ± 1.16^b^	20.21^b^	21.37 ± 1.36^b^
GLS 11	5.16^c^	5.22 ± 0.41^c^	4.26^c^	5.01 ±± 0.25^c^

*a,b,c – means with the same letter are not significantly different.

Once the optimal PLE conditions for recovering GLS from PC were identified, we decided to conduct a comparison between PLE and our previously optimized method based on USAE (Pagliari et al., 2022a). As expected, the pressurized liquid extraction proved to be the most efficient techniques for glucosinolates recovery, in fact, using this method, the recovery of the three GLSs increased by approximately 690 % (GLS9), 643 % (GLS10), and 801 % (GSL11) compared to the USAE procedure. However, the use of PLE requires highly qualified personnel and a high initial equipment cost. The results of the recovery of GLSs from the PC showed a high extraction efficiency of green techniques compared to the ISO technique.

### 3.3 Purification of the GLSs by solid phase extraction

Considering the good content of GLSs in PC it was decided to purify the extract by SPE to test the potential biological activity of an extract rich in GLSs.

**Fig. 3:**
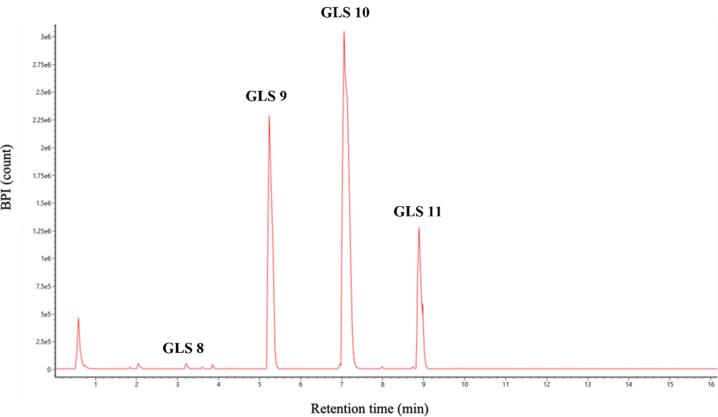
UPLC full MS chromatograms of pressurized liquid extraction after purification by solid phase extraction.

**Fig. 4:**
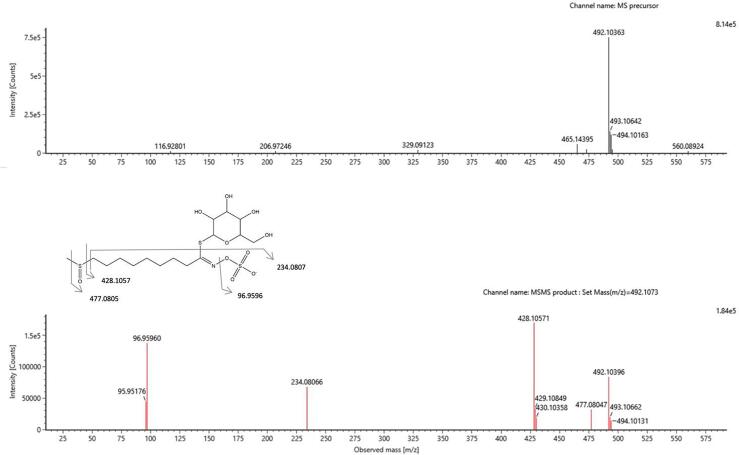
Full mass, ms/ms with fragmentation pathway of glucohirsutin (GLS8)in negative ion mode.

Both wash and elution fractions were collected, and each was analysed by UPLC-HRMS-DAD to detect the presence of GLSs and to verify the purity of the SPE extracts. Fig. 3 shows the HRMS chromatographic profiling of the elution fractions and crude extract. Chromatographic analysis shows the selective interaction of the negatively charged GLSs in the basic state with the NH_4_^+^ cartridges compared to the neutral phenolic compounds, which can not interact with the positive charges of the phase. Due to the concentration capacity of SPE procedure, the presence of new minority peaks in the purified PLE extract has been detected. For these reasons, a data dependent HRMS analysis was performed to tentatively identify the compounds detected. Among all these compounds, the peak at a retention time of 3.80 min with an accurate mass of 492.1036 *m*/*z* showed the specific fragmentation pattern of aliphatic glucosinolates according to MS/MS analysis (Fabre et al., 2007, Glauser et al., 2012). Specifically, the 477.0805 *m*/*z* production corresponds to the loss of a methyl (-15 Da), while the 234.0807 *m*/*z* ion corresponds to the fragment remaining after the loss of thioglucose and methyl sulfoxide and the presence of the 96.9596 *m*/*z* fragment corresponding to the sulphate group (Fig. 4). Based on this fragmentation pattern the glucohirsutin (GLS8) compound was tentatively assigned. To the best of our knowledge, this is the first time that GLS8 has been identified in the camelina sativa species.

### 3.4 Predictive bioactivities of the purified extract

The evaluation of the general biological potential of the four specific GLSs identified in C. sativa extract was initially investigated in silico using Prediction of activity spectra for substance (PASS) on-line software. The analysis was obtained by entering the simplified molecular input line entry Simplified Molecular Input Line Entry System (SMILES) strings of each molecule into the software and obtaining in response a table containing the probable activity (PA) and probable inactivity (PI) values with a related biological activity variability between 0.000 and 1.000. Biological predictions with PA > 0.700 show the greater potential biological activity.

The results reported in Table S2 show probable chemotherapeutic activity, in particular apoptosis agonist, chemopreventive and antineoplastic effects with Pa > 0.9 are predicted depending on their structure, indicating a very high probability results being reliable. These results are in agreement with the few data reported in literature regarding the activity of GLSs (Cabello-Hurtado et al., 2012). Hence, to investigate the biological activity of GLSs in purified PLE extract which was predicted using in silico analysis, spectrophotometric assays and in vitro cell analysis were conducted.

### 3.5 Scavenging effect of purified glucosinolates extract

Oxidative stress is one of the primary mechanisms involved in the onset and progression of non-communicable diseases such as cancer (Seyedsadjadi and Grant, 2021, Hariri and Ghiasvand, 2016). Oxidative stress is caused by the production of reactive oxygen species (ROS) and reactive nitrogen species (RNS). In physiological conditions, the production of radicals is minimal and is counteracted by endogenous cellular mechanisms. However, exogenous or endogenous factors can increase oxidative stress and impair the activity of cellular oxidoreductive processes (Borsoi et al., 2023). This disruption in the balance between radical production and endogenous mechanisms may contribute to the development and progression of various diseases including cancer. In this context, the antioxidant activity of natural molecules consumed through the diet play a crucial role in the prevention of pathological conditions. The predicted anticancer and chemopreventive activities of GLSs may be attributed to their potential antioxidant activity. Several studies have been reported the direct and/or indirect antioxidant capacity of GLSs, although their properties exhibit significant variations depending on their specific chemical structure. Hence, it was decided to evaluate the ability of the main GLSs identified in camelina sativa expeller to directly neutralise ROS and RNS. Scavenging activity was studied using three spectrophotometric assays (ABTS, ORAC and RNS). The ABTS assay showed a TE value of 69.77 ± 2.78 µg/mL EXT for the purified camelina extract, indicating a relatively low of capacity to neutralise ROS. This low capacity to neutralise ROS was also confirmed by the ORAC assay, which showed an IC50 value of percentage inactivation of 7.75 ± 0.22 µg/mL EXT, which was higher than the IC50 of ascorbic acid used as a positive reference (1.06 ± 0.07 µg/mL EXT). Regarding the activity against nitric radicals, a better neutralising effect was observed with an IC50 of 902.07 ± 19.35 µg/mL EXT much closer to that of ascorbic acid 842.18 ± 10.25. This suggests a better capacity of the purified extract to neutralise RNS. Based on the obtained result, the GLSs-enriched extract indicates a reduced direct antioxidant capacity, with enhanced efficiency against RNS radicals. These results agree with our previous study, which observed an increase in oxidative stress following its administration in tumour cells (Pagliari et al., 2022a). These results, combined with literature data, suggest an indirect antioxidant capacity, capable of stimulating the production of antioxidant enzymes in healthy cells, but not directly neutralise radical species.

### 3.6 Bioaccessibility and bioavailability of GLSs

To evaluate the potential application of GLSs-enriched extract as a health-promoting ingredient for dietary supplements, it was decided to verify the stability of GLSs following the gastrointestinal digestion simulation. INFOGEST is widely used for bioaccessibility studies of phytochemical compounds such as polyphenols, methylxanthines and numerous secondary metabolites, as well as for observing the digestive fate of proteins, lipids and carbohydrates (Brodkorb et al., 2019, Minekus et al., 2014). The results obtained showed an influence of the digestive process on the degradation of the main GLSs. This finding is in line with the literature, which shows that GLSs are molecules with reduced stability, sensitive to the action of pH and enzymes. Specifically, after the intestinal digestion step, the concentrations of GLS9 GLS10 and GLS11 remained at 32.24 ± 2.15, 50.03 ± 3.67 and 11.53 ± 0.99 µg/mg EXT, respectively, with a bioaccessibility of 28 % (GLS9), 26.80 % (GLS10) and 15.70 % (GLS11). However, in addition to the bioaccessibility of the molecules, it is also important to assess their bioavailability, or the percentage of the compound that is passively absorbed across the gastrointestinal barrier. Specifically, a bioavailability of 0 %-3.45 % has been calculated, indicating an inability to cross the intestinal barrier. This may be due to the structure of the molecule, which has a high molecular weight resulting in low permeability through the cell membrane. Further in-depth studies, perhaps using in vitro cellular systems to simulate the gastrointestinal barrier and evaluate its possible active absorption via transporters rather than the development of a suitable formulation to facilitate its passage, will therefore be required.

## 4 Conclusions

This study evaluated the efficient extraction of glucosinolates from camelina sativa by-product using pressurized liquid extraction. Through systematic optimization using an experimental design we have successfully identified the key parameters that maximize the yield of bioactive compounds, reducing the environmental impact. In detail, the optimized conditions extrapolated by Box-Behnken design were: EtOH 65 %, temperature 70 °C, 6 cycles, and static duration 2 min. After the identification of optimal extraction conditions for GLSs recovery, a comparative analysis of various extraction techniques (ISO and USAE) reveals that the optimized PLE extract has the highest GLSs recovery by reducing the time and organic solvent used. This result not only highlights the efficacy of the PLE method but also underscores its advantages in terms of sustainability, align with the principles of green chemistry. Following the optimization of extraction conditions, a selective purification procedure employing SPE have been used to selectively concentrate the GLSs and concurrently eliminate the interfering compounds. By employing this purification procedure which produced an GLS-rich extract the glucohirsutin, a previously unidentified glucosinolate in camelina sativa was discovered. Finally, the antioxidant activities and simulated digestion of the GLS-rich extract were investigated. The obtained extract exhibited significant potential for application in the food and pharmaceutical industries contributing to the growing demand for natural bioactive compounds with health-promoting benefits.

## Funding

This project was funded under the National Recovery and Resilience Plan (NRRP), Mission 4 Component 2 Investment 1.3 - Call for tender No. 341 of 15 March 2022 of Italian Ministry of University and Research funded by the European Union’s Next Generation EU; Project code PE00000003, Concession Decree No. 1550 of 11 October 2022 adopted by the Italian Ministry of University and Research, CUP D93C22000890001, Project title “ON Foods - Research and innovation network on food and nutrition Sustainability, Safety and Security – Working ON Foods”.

## CRediT authorship contribution statement

**Stefania Pagliari:** Writing – original draft, Investigation, Data curation. **Gloria Domínguez‐Rodríguez:** Formal analysis. **Alejandro Cifuentes:** Writing – original draft, Supervision. **Elena Ibáñez:** Writing – original draft, Supervision. **Massimo Labra:** Supervision, Funding acquisition, Conceptualization. **Luca Campone:** Writing – review & editing, Supervision, Funding acquisition, Conceptualization.

## Declaration of competing interest

The authors declare that they have no known competing financial interests or personal relationships that could have appeared to influence the work reported in this paper.

## Data Availability

Data will be made available on request.
