# Supplementary material for: Pressurized liquid extraction of glucosinolates from Camelina sativa (L.) Crantz by-products: Process optimization and biological activities of green extract
Source: Food Chem X. 2024 Mar 24;22:101324. doi: 10.1016/j.fochx.2024.101324 (PMC10999800; doi:10.1016/j.fochx.2024.101324)
Supplement: Supplementary data 1 [file mmc1.docx]

**Supplementary Material**

**Pressurized green liquid extraction of glucosinolates from *Camelina sativa* (L.) Crantz by-products: Process optimization and biological activities of green extracts.**

Stefania Pagliari ^1^, Gloria Domínguez‐Rodríguez ^3^, Alejandro Cifuentes ^3^, Elena Ibáñez ^3^, Massimo Labra ^1,2^, Luca Campone*^1,2^

^1^ Department of Biotechnology and Biosciences, University of Milano-Bicocca, Milan, Italy

^2^ NBFC, National Biodiversity Future Center, 90133 Palermo, Italy

^3^ Foodomics Laboratory, Instituto de Investigación en Ciencias de la Alimentación (CIAL, CSIC-UAM), Nicolás Cabrera 9, Campus de Cantoblanco, 28049 Madrid, Spain

*Corresponding author e-mail address: [luca.campone@unimib.it](mailto:luca.campone@unimib.it)


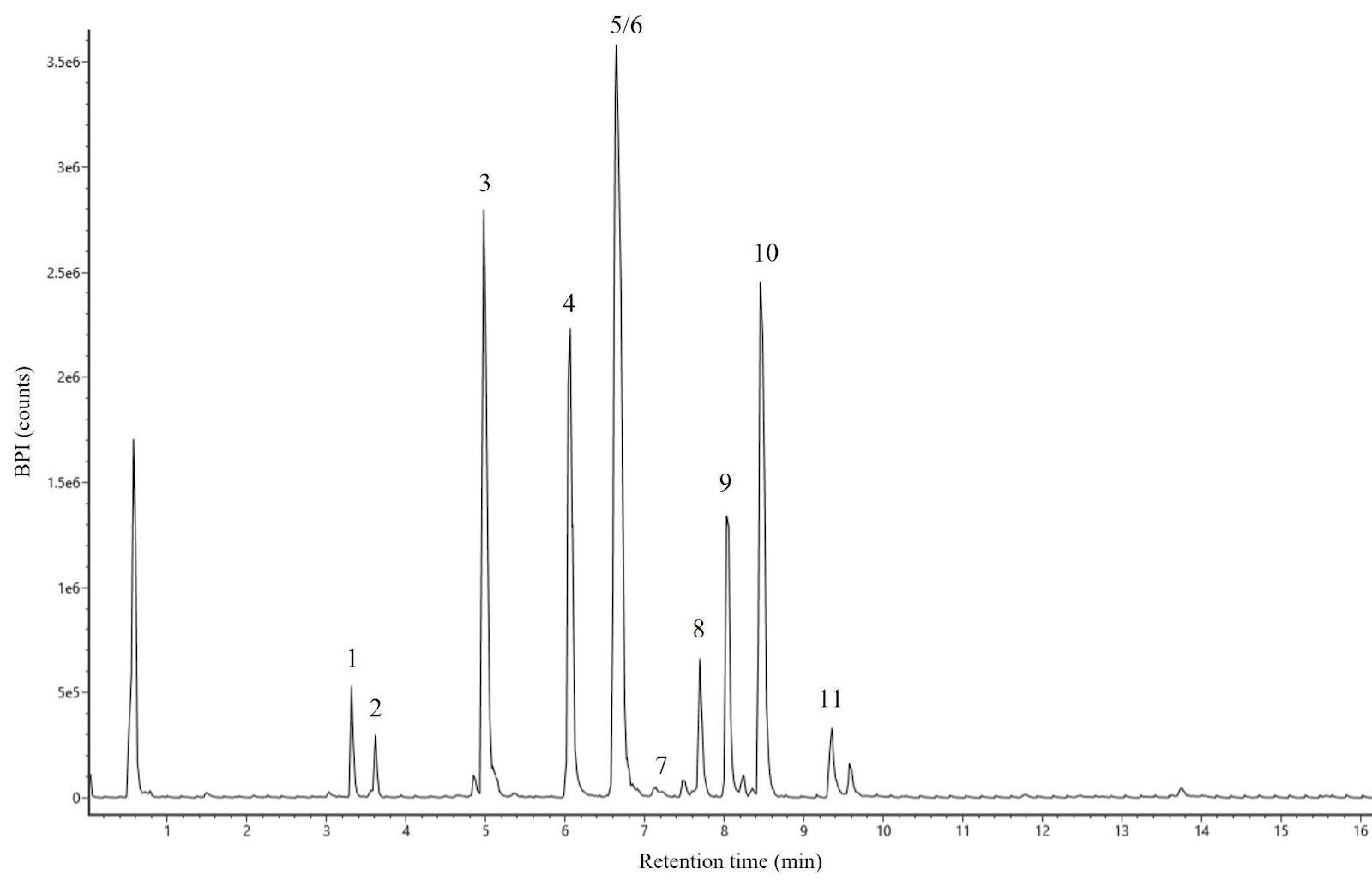


**Figure S1** Representative chromatogram of *Camelina sativa* PLE extract obtained by UPLC-MS in negative ionization mode.

**Table S1 Equation of regression model by DoE**

| **Equation number** | | **Response** | **Equation** |
| --- | --- | --- | --- |
| 1 | GLS9 | | - 43310,6 + 808,725 x EtOH – 4,70156 x EtOH^2^ – 45,4437 x EtOH x cycle |
| 2 | GLS10 | | - 87226,7 + 1706,12 x EtOH – 9,8675 x EtOH^2^ – 91,9125 x EtOH x cycle |
| 3 | GLS11 | | - 8404,8 + 238,079 x EtOH – 1,51104 EtOH^2^ – 14,575 x EtOH x cycle |

**Table S2**: Main potential biologic activities predicted using Pass online based software.

| **Glucosinolates** | **Predicted bioactivities** | **Pa** | **Pi** |
| --- | --- | --- | --- |
| Glucohirsutin (GLS8) | Chemopreventive  Apoptosis agonist  Antineoplastic | 0.985  0.928  0.833 | 0.001  0.004  0.008 |
| Glucoarabin (GLS9) | Chemopreventive  Apoptosis agonist  Antineoplastic  Immunostimulant | 0.985  0.928  0.833  0.577 | 0.001  0.004  0.008  0.026 |
| Glucocamelinin (GLS10) | Chemopreventive  Anticarcinogenic  Apoptosis agonist  Antineoplastic | 0.947  0.907  0.905  0.782 | 0.002  0.002  0.004  0.014 |
| Homoglucocamelinin (GLS11) | Chemopreventive  Anticarcinogenic  Apoptosis agonist  Antineoplastic | 0.947  0.907  0.905  0.782 | 0.002  0.002  0.004  0.014 |
